# Phytochemical Analysis and Multifaceted Biomedical Activities of *Nitraria retusa* Extract as Natural Product-Based Therapies

**DOI:** 10.3390/life14121629

**Published:** 2024-12-09

**Authors:** Manal M. Khowdiary, Zinab Alatawi, Amirah Alhowiti, Mohamed A. Amin, Hussam Daghistani, Faisal Miqad K. Albaqami, Mohamed Ali Abdel-Rahman, Ahmed Ghareeb, Nehad A. Shaer, Ahmed M. Shawky, Amr Fouda

**Affiliations:** 1 Department of Chemistry, Faculty of Applied Science, Lieth Collage, Umm Al-Qura University, Makkah 24382, Saudi Arabia; mmkhowdiary@uqu.edu.sa (M.M.K.); nashaer@uqu.edu.sa (N.A.S.); 2 Department of Family and Community Medicine, Faculty of Medicine, University of Tabuk, Tabuk 47512, Saudi Arabia; zalatawi@ut.edu.sa (Z.A.); aalhowiti@ut.edu.sa (A.A.); 3 Department of Botany and Microbiology, Faculty of Science, Al-Azhar University, Nasr City, Cairo 11884, Egypt; mamin7780@azhar.edu.eg (M.A.A.); mohamedali@kyudai.jp (M.A.A.-R.); 4 Department of Clinical Biochemistry, Faculty of Medicine, King Abdulaziz University, Jeddah 21589, Saudi Arabia; hmdaghistani@kau.edu.sa; 5 Regenerative Medicine Unit, King Fahd Medical Research Center, King Abdulaziz University, Jeddah 21589, Saudi Arabia; 6 Biology Department, Faculty of Science, Islamic University of Madinah, Madinah 42351, Saudi Arabia; falbaqami@iu.edu.sa; 7 Botany and Microbiology Department, Faculty of Science, Suez Canal University, Ismailia 41522, Egypt; 8 Science and Technology Unit (STU), Umm Al-Qura University, Makkah 21955, Saudi Arabia; amesmail@uqu.edu.sa; 9 School of Nuclear Science and Technology, University of South China, Hengyang 421009, China

**Keywords:** plant extracts, *Nitraria retusa*, antioxidant activity, cytotoxicity, anti-diabetic, antimicrobial activity

## Abstract

This study examined the phytochemical profile and biomedical activities of *Nitraria retusa*, a halophytic and drought-resistant shrub. HPLC analysis showed gallic acid (1905.1 μg/g), catechin (1984.1 μg/g), and ellagic acid (2671.1 μg/g) as the primary constituents, while FT-IR analysis revealed a complex organic profile with significant functional groups. The extract demonstrated strong antioxidant activity in DPPH assays, outperforming ascorbic acid (IC_50_ = 18.7 ± 1.0 μg/mL) with an IC_50_ of 16.4 ± 4.4 μg/mL. It demonstrated specific antiproliferative effects on cancer cell lines as it showed selective cytotoxicity against cancer cell lines; normal WI38 cells were largely unaffected, showing 50.0% viability at 125 μg/mL. The most sensitive cell line was Caco2, which showed 50.0% viability at 125 μg/mL. Anti-diabetic properties were exhibited by means of inhibition of α-amylase (IC_50_ = 68.2 ± 4.2 μg/mL) and α-glucosidase (IC_50_ = 22.8 ± 3.3 μg/mL). Additionally, antimicrobial activity was observed to be broad-spectrum, and it was most effective against *E. coli* (32.6 mm inhibition zone at 400 μg/mL) and *Penicillium glabrum* (35.3 mm at 400 μg/mL). These findings highlight the potential of *N. retusa* in developing plant-based therapeutic approaches.

## 1. Introduction

Over the past ten years, there has been a significant increase in the popularity of traditional medicine practices worldwide. The research groups and large pharmaceutical companies have focused on the plant kingdom in their quest for novel drugs and physiologically active compounds [1]. Growing interest in natural remedies has led to more research on medicinal plants, as these plants are rich in substances that may have therapeutic properties. Phenolic compounds are the most significant antioxidants among the plant metabolites that have been isolated from medicinal plants [2]. Their significance stems from their ability to both scavenge free radicals and block the processes that lead to the synthesis of these compounds. The extraction technique for each of these compounds is dependent upon their respective chemical structures, which range from simple to highly polymerized forms [3,4].

Significantly, the complex antioxidant profiles of various plant species can be influenced by the varying concentrations of phenolic acids, flavonoids, flavanols, anthocyanins, and tannins found in plant materials. This structural diversity is responsible for the varying antioxidant potencies found in various compounds derived from plants, as well as influencing the necessary extraction techniques [5]. Phenolic compounds exhibit a range of physicochemical properties because of their diverse structures, which are especially responsible for their solubility in various solvents. Because solvent polarity greatly affects the extraction efficiency of particular compounds, this variability makes it difficult to develop a universal extraction technique for all phenolic compounds from plants [6,7].

Polyphenolic compounds are efficiently extracted from plant materials using polar solvents. Commonly employed solvents for this purpose are (C_2_H_5_)_2_O, CH_3_OH, C_2_H_5_OH, (CH_3_)_2_CO, and H_2_O. More total polyphenols are produced by CH_3_OH, whereas (CH_3_)_2_CO is especially useful for high molecular weight flavonoids. A plant’s origin and the chemical composition of the target compounds determine the best extraction technique [8]. The extraction time, temperature, solvent ratio, and repeat cycle can all be adjusted as needed. This method can be applied to various plant-based sources, making it valuable for phytochemical research and natural product isolation in a range of fields [9].

Halophytes, salt-tolerant plants, are abundant producers of secondary metabolites with high economic value; their quantity and structural diversity frequently outweigh that of glycophytes. These substances are valuable for pharmacognosy and functional foods because they can act singly or in combination [10]. Despite their potential, many halophyte applications are still in the research and development phase. The genus *Nitraria* comprises of salt-tolerant shrubs that thrive in both saline and dry conditions. One of its species, *Nitraria retusa* Asch, is classified under the Nitrariaceae family [11]. This broad family, which includes shrubs, trees, and herbs, is made up of 27 genera and 270 species. *Nitraria* species are well known for their resilience and global distribution, especially in the arid and salt marshy regions of central Asia [12].

Great attention has been paid to the genus *Nitraria* because of its wide range of traditional applications and rich phytochemical profile. Flavonoids constitute the majority of the polyphenols found in these plants [13]. A key component of these flavonoids, isorhamnetin, has strong biological activities, particularly antiproliferative and antioxidant properties. This phytochemical diversity contributes to a broad range of therapeutic uses across various *Nitraria* species [14,15]. In traditional medicinal practices, the fruits are used both as food and to treat hypertension and gynecological imbalances. *Nibirica* fruits, for instance, are well known for their ability to treat hypertension [16]. The leaves of *N. tangutorum* are used to prevent arrhythmia and neuropathy, and the fruits are used to treat colds, dyspepsia, stomachaches, and chronic fatigue syndrome [17,18]. Traditionally, gastrointestinal disorders ranging from poisoning to discomfort in the abdomen are treated with a decoction made from fresh *N. retusa* leaves in North African medicine. It has also been discovered that the adaptable leaves of *Nitraria* species are beneficial as beverage supplements and therapeutic plasters. Additionally, the plant’s ashes have medicinal properties as well and can be used to treat infected wounds [19].

*Nitraria retusa* has become a plant of great scientific interest because of its wide range of bioactive compounds and potential for therapeutic use. Analysis of leaves has shown a high content of flavonoids, especially different types of isorhamnetin. These compounds are responsible for the plant’s strong antioxidant capabilities, as demonstrated by the plant’s strong neutralization of free radicals in laboratory tests [20]. Besides its antioxidant qualities, the plant has other therapeutic benefits. Experiments with leaf extracts have not only shown protective effects against genetic damage but also shown promising results in inhibiting the growth of certain cancer cell lines, like Caco-2 and K562 [21]. These findings align with larger-scale research demonstrating the antimicrobial and anti-mutagenic qualities of the leaves, as well as their capacity to induce programmed cell death in cancer cells [21]. *N. retusa* leaves also have additional medical applications. Studies on fruit extracts have shown that they can shield rat kidneys from the fungicide penconazole’s toxicity [22]. Moreover, recent research has demonstrated that leaf extracts treated with chloroform possess immunomodulatory and anti-tumor properties; these effects are attributed to the presence of palmitic acid and β-sitosterols [23].

The main goal of this study was to perform a comprehensive investigation into the phytochemical composition and biological activities of *Nitraria retusa*. By investigating the plant extract’s antioxidant, anticancer, anti-diabetic, and antimicrobial activities using several in vitro tests, the study elucidated the plant extract’s potential therapeutic applications. The study aimed to characterize the extract’s chemical profile using HPLC analysis, evaluate its capacity to scavenge free radicals using the DPPH assay, investigate its cytotoxic effects on normal and cancerous cell lines (WI38, Caco2, and MCF7) through the MTT assay, explore its anti-diabetic potential by measuring α-amylase and α-glucosidase inhibition, and determine its antimicrobial efficacy against both bacterial (*S. aureus* and *E. coli*) and fungal (*Candida albicans* and *Penicillium glabrum*) strains using the agar well diffusion method.

## 2. Materials and Methods

### 2.1. Preparation and Extraction of N. retusa Leaf Extract

**Figure 1:**
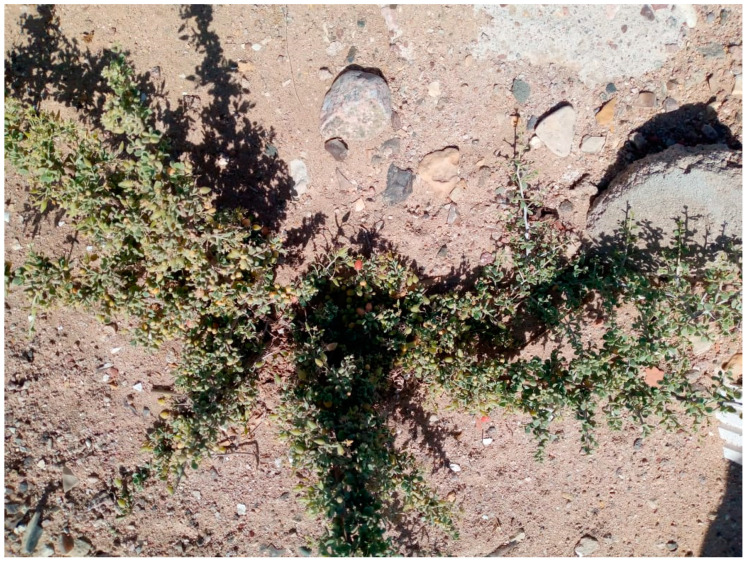
*N. retusa* plant collected from Wadi Hagul, Eastern Desert, Egypt.

*N. retusa* leaves were collected from Wadi Hagul (29°55′38.91″ N and 32°11′37.12″ E), Eastern Desert, Egypt (Figure 1). The sample collection site is characterized by high temperature, rare rainfall, and high evaporation rate. The collected samples were prepared in three steps: they were washed well under running tap water, dried in the shade at room temperature, and then ground into a powder. In a soxhlet extractor, 100 g of this powder and 250 mL of ethyl acetate were mixed for the extraction process [24,25]. After that, the extract was dried and filtered at 45 °C using a rotary evaporator (Heidolph Instruments GmbH & Co. KG, Schwabach, Germany). For use in later studies, the final yield was stored in DMSO [26].

### 2.2. FT-IR and HPLC Analyses of Nitraria retusa Extract

To prepare the extract for FT-IR analysis, 2.0 mg of dried *N. retusa* extract and 200 mg of KBr were thoroughly blended. After that, the mixture was compressed to create a thin, transparent disk that could be examined under a microscope using a Bruker Vector 22 Spectrophotometer (FTIR-UNIT, Coventry, UK). A wavenumber range of 400 to 4000 cm^−1^ was used to record the FT-IR spectrum [27].

**Figure 2:**
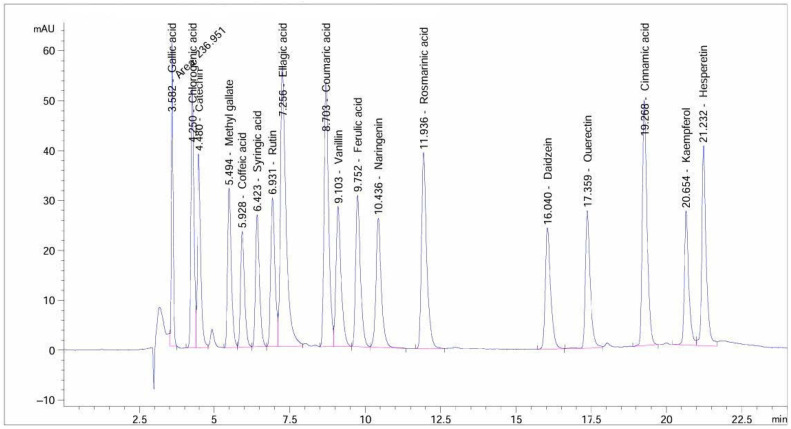
HPLC standards used in the current investigation.

To separate and identify the chemical components of the extract, HPLC analysis was performed. This was accomplished by using an Agilent 1260 HPLC system with a Zorbax Eclipse Plus C8 column (4.6 × 250 mm, 5 μm particle size). The mobile phase used in the analysis was made up of water (solvent A) and CH_3_CN with 0.05% CF_3_COOH (solvent B). In the 24 min linear gradient elution program, 82% water (solvent A) was used for one minute, then 75% over ten minutes, and finally 60% over seven minutes. After that, the concentration rose once more to 82% over the next four minutes and remained there for the final two minutes. Throughout the run, the column temperature was maintained at 40 °C and the flow rate was kept constant at 0.9 mL/min [28]. A variable wavelength detector with a 280 nm setting and a 5 μL sample injection volume was used for detection. The extract’s components could be effectively separated and their chemical composition analyzed using this optimized method. Validation of HPLC analysis was achieved by an external partner, and their chart has been included (Figure 2; Table S2).

### 2.3. Antioxidant Analysis of Nitraria retusa via DPPH Reduction

A solution of 0.1 mM DPPH in C_2_H_5_OH was prepared. Three milliliters of different ethanol-soluble *Nitraria retusa* extract (concentrations ranging from 3.9 to 1000 μg/mL) were mixed with one milliliter of this solution. The mixture was vigorously shaken and left at room temperature for 30 min. Next, a UV-VIS Milton Roy spectrophotometer (Thermo Fisher Scientific in Waltham, MA, USA) was used to measure absorbance at 517 nm. Three duplicates of the experiment were carried out, with ascorbic acid acting as the reference standard [29]. With the use of a log dose-inhibition curve, the IC_50_ value, which indicates the concentration of the plant extract needed to inhibit 50% of DPPH free radicals, was determined.

(1) $$\mathrm{DPPH}\;\mathrm{Scavenging}\%=\frac{O.D._{\mathrm{control}}-O.D._{\mathrm{treatment}}}{O.D._{\mathrm{control}}}\times 100$$

### 2.4. Cytotoxicity of Nitraria retusa Extract

An MTT assay was used to evaluate the plant extract’s cytotoxicity against several cell lines, including Mcf7 (breast cancer), Caco2 (colorectal adenocarcinoma), and WI38 (normal lung fibroblasts), all of which were acquired from VACSERA in Cairo, Egypt. The experiment’s main goal was to determine IC50 values relative to a reference dose of cisplatin. In the beginning, 96-well plates were seeded with 1 × 10^4^ cells per well and 100 μL of growth media for 24 h at 37 °C with 5% CO_2_. Subsequently, the cells were incubated until a monolayer sheet formed. It was then washed twice with maintenance RPMI media with serum (2%). Following this step, different concentrations of the plant extract (31.25–1000 μg/mL) were used for cell treatment for 48 h. Three wells were treated with DMSO (control) under the same conditions. At the end of the incubation period, excess RPMI media was removed, and each well was treated with 50 µL of MTT solution (5 mg dissolved in 1 mL of PBS) for 4 h at 37 °C. After that, each well received 100 µL of 10% DMSO (after discarding excess MTT solution) for 30 min under shaking conditions to dissolve the formazan crystal. Using an ELISA reader (TECAN, Inc., Männedorf, Switzerland), the absorbance of the formed color was detected at a wavelength of 570 nm, and cell viability was calculated using the following formula [30,31]:

(2) $$Cell\;viability\;\%=\frac{Ab_{treatment}}{Ab_{control}}\times 100$$

where *Ab* is the absorbance.

### 2.5. Anti-Diabetic Potential of Nitraria retusa Extract

#### 2.5.1. α-Amylase Inhibition Assay Using DNS Method

The 3,5-dinitrosalicylic acid method (DNS) was used to perform the α-amylase inhibition assay. To attain concentrations ranging from 1.9 to 1000 μg/mL, the plant extract was first dissolved in 10% DMSO and then diluted in a buffer (0.02 M Na_2_HPO_4_, 0.006 M NaCl, pH 6.9). To perform the assay, 200 μL of α-amylase solution (2 units/mL) was mixed with the same volume of extract and then incubated at 30 °C for 10 min. Then, 200 μL of a 1% starch solution was added, and the mixture was incubated for three minutes. A 200 μL volume of DNS reagent (consisting of 12 g KNaC_4_H_4_O_6_·_4_H_2_O, 8.0 mL 2 M NaOH, and 20 mL 96 mM DNS solution) was added to stop the reaction. After 10 min of boiling at 85 to 90 °C, this mixture was allowed to cool to room temperature before being diluted with 5 mL of distilled water [32]. Finally, absorbance was measured at 540 nm using a UV–visible Biosystem 310 spectrophotometer (BioSystems S.A. in Barcelona, Spain).

(3) $$\mathrm{Inhibition}\;\%=\frac{Ab_{control}-Ab_{treatment}}{Ab_{control}}\times 100$$

where *Ab* is the absorbance.

#### 2.5.2. In Vitro α-Glucosidase Inhibition

The Pistia-Brueggeman and Hollingsworth method was modified to assess the α-glucosidase inhibitory activity of *Nitraria retusa* extract [33]. A 50 μL volume of plant extract (concentration ranging from 1.97 to 1000 μg/mL) was combined with 125 μL of 0.1 M phosphate buffer (pH 6.8) and 10 μL of α-glucosidase solution (1 U/mL) for the analysis. This mixture was incubated at 37 °C for 20 min. The reaction was then initiated by adding 20 μL of 1 M pNPG substrate and incubated for a further 30 min. A 50 μL volume of 0.1 N Na_2_CO_3_ was added to terminate the reaction. The final absorbance was measured at 405 nm using a Biosystem 310 plus spectrophotometer (BioSystems S.A. in Barcelona, Spain). The α-Glucosidase inhibition percentages were calculated using Equation (3).

### 2.6. Antimicrobial Properties of Nitraria retusa Extract

Using a variety of microbial strains, including *E. Coli* (ATCC 8739), *S. aureus* (ATCC 6538), *Penicillium glabrum* (OP694171), and *Candida albicans* (ATCC 10221), the antimicrobial activity of *Nitraria retusa* extract was assessed. The extract’s inhibitory effects were evaluated at concentrations ranging from 12.5 to 400 μg/mL using the agar well diffusion method. In this experiment, bacterial strains were refreshed on nutrient agar plates overnight at 35 ± 2 °C. Then, individual colonies were reinjected into Mueller Hinton agar plates, where wells (0.6 mm diameter) were created. Various concentrations of the extract (100 µL) were added to each well. Plates were refrigerated for an hour to facilitate diffusion and then incubated for twenty-four hours at 35 ± 2 °C [34].

Sabouraud dextrose agar was utilized for fungal strains and was incubated for 48 h. As a negative control, DMSO was used in each plate at one concentration, whereas for the positive control, gentamicin for bacterial strains and fluconazole for fungal strains were applied at the same concentrations under the same conditions. The minimum inhibitory concentration (MIC), which is the lowest concentration that produced an inhibition zone, was used to measure inhibition zones in millimeters [35]. All experiments were conducted in triplicate to ensure reliability.

### 2.7. Statistical Analysis

SPSS (version 18, SPSS Inc., Chicago, IL, USA) was utilized for data analysis. The Shapiro–Wilk test, Equal Variance Test, and One-way Analysis of Variance (ANOVA) were performed. Following ANOVA, post-hoc tests were conducted.

## 3. Results and Discussion

### 3.1. Nitraria retusa Metabolites Profiling and Antioxidant Assessments

Based on FT-IR spectral analysis, the extract of *Nitraria retusa* exhibited a complex organic composition comprising multiple important functional groups (Figure S1; Table S1). The spectrum showed a strong and broad peak at 3353 cm^−1^, which was correlated with the stretching O-H group of alcohols or carboxylic acids [36]. This observation was further supported by two peaks, one at 1675 cm^−3^ and the other at 1273 cm^−3^, corresponding to stretching C=O and C-O, respectively, which point to the functionality of carboxylic acids. Aliphatic components, which contain branched or straight chains of hydrogen and carbon atoms such as alkanes, alkenes, and alcohols, were also present in the plant extract. As shown, the medium-intensity peaks at 2819 and 2851 cm^−1^ indicated the alkyl C-H stretching. Aromatic structures were also present, as indicated by medium-intensity peaks at 1600 cm^−1^ (C=C stretch) and weak-intensity peaks in the 720–630 cm^−1^ range (C-H bend). Moreover, a medium peak at 1128 cm^−1^ suggested the presence of additional C-O bonds, possibly from alcohol groups, while medium-intensity peaks in the 1000–900 cm^−1^ range could indicate alkene (=C-H) bending [37]. These observations supported *Nitraria retusa* extract as a rich source of diverse organic compounds, including both phenolic acids and flavonoids.

**Figure 3:**
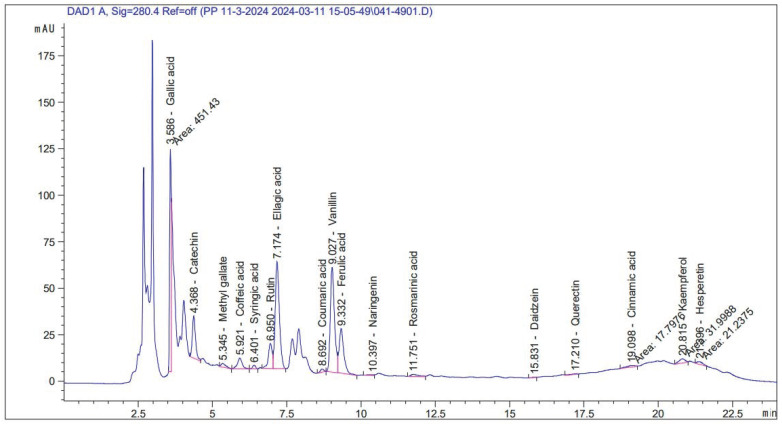
Phytochemical fingerprint of *Nitraria retusa* extract based on HPLC analysis.

To better understand the composition of the extract and to further clarify the specific compounds contributing to these spectral features, HPLC analysis was carried out. Using this technique, individual compounds can be separated, identified, and quantified, providing a more accurate assessment of the phytochemical profile of the extract. The *Nitraria retusa* extract’s HPLC chromatogram and associated data table (Figure 3 and Table 1) revealed several significant and noteworthy bioactive compound diversity within the extract. Various well-resolved peaks could be seen in the chromatogram, which suggested that different compounds had been effectively identified using the HPLC method (Figure 3). According to quantification results, the extract had notable concentrations of ellagic acid (2671.1 µg/g), catechin (1984.1 µg/g), and gallic acid (1905.1 µg/g), followed by significant amounts of vanillin (1083.5 µg/g) and rutin (1010 µg/g) (Table 1).

These main ingredients were enhanced by a variety of additional substances. Naringenin (27.3 µg/g), quercetin (14.3 µg/g), kaempferol (119.1 µg/g), and hesperetin (52.7 µg/g) were among the flavonoids. Compounds that represented phenolic acids were ferulic acid (782.4 µg/g), coumaric acid (23.8 µg/g), syringic acid (49.6 µg/g), and caffeic acid (284.9 µg/g). Notably, there was no trace of chlorogenic acid (0.00 µg/g) in the sample. In addition, the extract included minor components that added to its overall phytochemical complexity, such as the phenolic ester methyl gallate (65.2 µg/g), the isoflavone daidzein (4.06 µg/g), and the phenylpropanoid cinnamic acid (16.7 µg/g) (Table 1).

The presence of these varied components in the *Nitraria retusa* extract reveals its multifaceted therapeutic potential. For instance, the presence of ellagic, catechin, and gallic acids as the main constituents of the plant extract exhibits the antioxidant properties of this extract. Moreover, these components showed different biological activities, such as anti-inflammatory, anti-carcinogenic, and antimicrobial activity, which increase the potential applications of *N. retusa* extract [21,38]. In addition to the antioxidant activity of rutin and vanillin, it exhibits hepato- and neuroprotective potentials, alleviates oxidative stress, and improves vascular health [39]. Moreover, the presence of some flavonoid compounds, such as naringenin, quercetin, kaempferol, and hesperetin, even in minor quantities, has efficacy in alleviating oxidative stresses, preventing or reducing inflammation, and protecting against chronic diseases [15]. HPLC analysis exhibited the unique phenolic components of the *N. retusa* plant via the presence of ferulic acid, syringic acid, and caffeic acid. These phenolic components have various therapeutic activities, such as combating oxidative stress, as well as anti-diabetic, antimicrobial, antioxidant, and anticancer properties [38,40].

### 3.2. Antioxidant Activity

**Figure 4:**
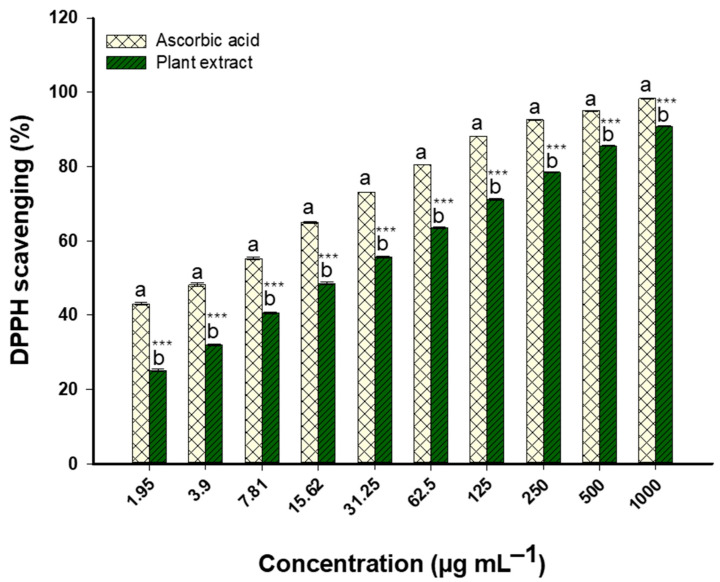
DPPH radical scavenging activity of *Nitraria retusa* extract vs. ascorbic acid (1.95–1000 μg/mL, n = 3). Different letters (a and b) on the bars indicate significant differences between treatments at the same concentration. *** *p* < 0.001 indicate significant differences from control group (*p* < 0.05, *p* < 0.01, and *p* < 0.001, respectively); (n = 3).

The antioxidant potential of the *Nitraria retusa* extract was then determined by evaluating its DPPH scavenging activity. Antioxidant activity increased in a concentration-dependent manner, according to the results, with the largest improvements obtained in the lower concentration range (1.95 to 62.5 μg/mL) and smaller incremental increases obtained at higher concentrations. The extract exhibited significant antioxidant capacity even at low concentrations, as evidenced by its 48.4% scavenging activity at 15.62 μg/mL. The extract was found to be effective, as evidenced by its lower IC50 value of 16.4 ± 4.4 μg/mL compared to ascorbic acid control (18.7 ± 1.0 μg/mL). Scavenging activity increased with concentration; at 31.25 μg/mL and above, levels exceeded 50% (Figure 4). Significant scavenging activities were achieved at concentrations of 125 μg/mL and above; at these levels, the extract demonstrated over 70% DPPH scavenging activity, peaking at 90.8% at 1000 μg/mL.

In accordance with our findings, the DPPH assay was used by Almasarwah et al. [41] to evaluate the antioxidant potential of *Nitraria retusa* extracts. Compared to the aqueous extract (1249.3 ± 233.5 µg/mL), the methanol extract showed a significantly lower IC_50_ value (958.2 ± 178.3 µg/mL). These findings suggest that *N. retusa*’s methanol extract has a stronger capacity to scavenge free radicals than its aqueous counterpart [41]. Similar findings were obtained by Rjeibi et al. [42], who reported that *N. retusa* leaf extracts (NRLP) had notable antioxidant qualities. With an IC_50_ of 2.7 mg/mL, the DPPH radical scavenging activity of NRLP peaked at 73.5% (3 mg/mL). Additionally, the extract showed moderate hydroxyl radical scavenging (IC_50_ = 2.42 mg/mL) and strong total antioxidant activity (IC_50_ = 2.03 mg/mL), demonstrating its broad antioxidant potential.

The synergistic effect of the extract’s diverse bioactive components, such as different flavonoids and phenolic acids, may be responsible for the strong antioxidant activity of *Nitraria retusa*. The extract exhibited a remarkable capacity to scavenge DPPH, surpassing ascorbic acid in this regard, with an IC_50_ value of 16.4 ± 4.4 μg/mL, as opposed to 18.7 ± 1.0 μg/mL for ascorbic acid. High concentrations of known antioxidants were found in the extract, including gallic acid (1905.1 µg/g), ellagic acid (2671.1 µg/g), and catechin (1984.1 µg/g). Additionally, significant amounts of vanillin and rutin were present. The plant’s complex antioxidant profile most likely evolved as an adaptive response to its demanding, harsh environment, which is marked by high salinity and drought conditions. *N. retusa* evolved to generate and accumulate these protective compounds in response to environmental stressors that cause oxidative stress. The plant’s ability to adapt not only allowed it to survive in challenging environments but also enhanced its antioxidant capacity, as evidenced by its increased performance in the DPPH test.

### 3.3. Selective Cytotoxicity of Nitraria retusa Extract Against Cancer Cell Lines

**Figure 5:**
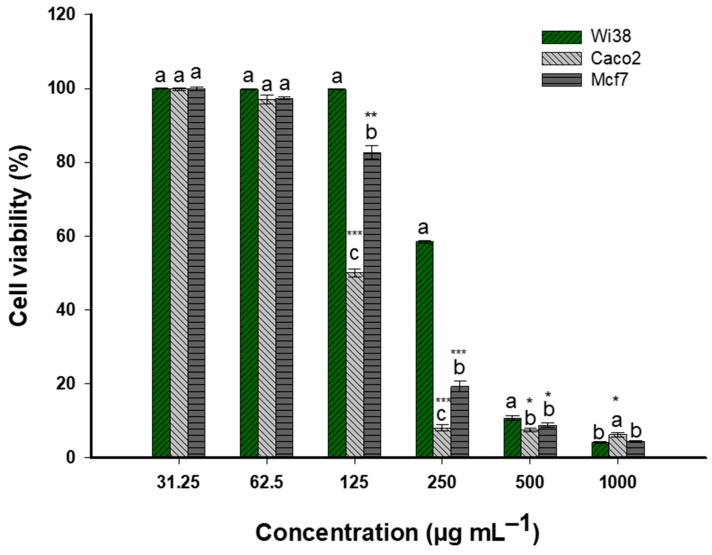
Dose-dependent cytotoxicity of plant extract on Wi38, Caco2, and Mcf7 cell lines. Data represents mean cell viability (%). Different letters (a, b, and c) on the bars at the same concentration indicate significant differences. *, **, and *** *p* < 0.001 indicate significant differences from control group (*p* < 0.05, *p* < 0.01, and *p* < 0.001, respectively); (n = 3).

*Nitraria retusa* extract demonstrated variations in sensitivity with a dose-dependent cytotoxic effect against the three cell lines, WI38, Caco2, and MCF7 (Figure 5). Significantly, the extract showed increased effectiveness against cancerous cell lines, especially Caco2, at moderate concentrations. All cell lines demonstrated high viability (>99%) at the lowest concentration (31.25 μg/mL), suggesting minimal toxicity. When the concentration reached 62.5 μg/mL, there was a small difference in viability between Caco2 and MCF7 cells (96.9% and 97.3%, respectively) and WI38 cells (99.768%).

A significant threshold was obtained at 125 μg/mL, where cell lines exhibited markedly different responses. Significantly, the viability of Caco2 cells dropped to 50.0%, whereas MCF7 cells demonstrated moderate sensitivity (82.6% viability), and WI38 cells remained mostly unaffected (99.8% viability). It seems that this concentration is essential for proving the extract’s specific cytotoxicity against Caco2 and other cancerous cell lines. All cell lines exhibited significant reductions in viability at 250 μg/mL, though to different degrees: the most affected cell line was Caco2 (8.0% viability), followed by MCF7 (19.2%) and WI38 (58.4%) (Figure 5). This highlights even more how much more lethal the extract is to cancerous cells than to healthy cells. All cell lines showed significantly decreased viability (<11%) at the highest concentrations (500 and 1000 μg/mL), indicating strong cytotoxic effects independent of the type of cell.

Researchers examined the cytotoxic effects of six fractions obtained from the methanolic extract of *Nitraria retusa* at 100 μg/mL on a variety of human cancer cell lines. Across several cell lines, especially MCF-7 and HEPG-2 cells, the n-hexane (N-He) fraction showed the strongest cytotoxicity, reducing their growth by 98.5 ± 0.7% and 96.0 ± 0.8%, respectively. Moreover, it demonstrated noteworthy activity against HCT-116, exhibiting 47.5 ± 0.9% inhibition. Specifically on A-549 and PC-3 cells, the dichloromethane (N-Dm) fraction showed moderate effects, with inhibition rates of 24.8 ± 0.4% and 20.1 ± 0.8%, respectively. In contrast, there was little cytotoxic activity in the n-butanol (N-Bu) and ethyl acetate (N-Ea) fractions [43].

The antitumoral and immunomodulatory properties of *N. retusa* leaf methanol extract were examined in a different study. For 21 days, 200 mg/kg bw of the extract was administered to Balb/c mice that had been implanted with B16-F10 cells. Significantly suppressing tumor growth, the extract also improved immune responses, as demonstrated by increased splenocyte proliferation, NK and CTL activity, and macrophage lysosomal activity. The authors proposed that the extract’s immunomodulatory qualities were responsible for its antitumoral effect [44].Another study investigated the biological effects of *N. retusa* leaf extracts on B16F10 melanoma cells as well as the phytochemical profile. Analysis of DNA fragmentation revealed that the extracts exhibited strong anti-proliferative activity after 48 h. Moreover, tyrosinase activity and melanogenesis were increased in the melanoma cells by all tested extracts [45]. Furthermore, the antitumoral effects of *N. retusa* leaf chloroform extract were investigated, with a particular focus on its two main constituents, palmitic acid and β-sitosterols. B16-F10 cell tumor-ridden BALB/c mice received intraperitoneal therapy for a duration of 21 days. The components of the extract improved immune responses, including cytotoxic T lymphocyte activities and increased splenocyte proliferation, and significantly inhibited tumor growth while protecting lung parenchyma. In addition, they reduced lipid peroxidation and enhanced cellular antioxidant capacity and macrophage lysosomal activity [23,46].

The rich polyphenolic content of the extract under study, especially that of gallic acid (GA), ellagic acid (EA), and catechin, as well as notable levels of flavonoids and phenolic acids, are responsible for the extract’s anticancer properties. These substances are well-known for their potent antiproliferative and antioxidant qualities; EA has been demonstrated to cause cell cycle arrest and apoptosis in several cancer cell lines. For example, in a study by Li and colleagues, (EA) showed strong anticancer properties against cells that cause cervical cancer. After treating HeLa, SiHa, and C33A cell lines with EA, the researchers reported a significant decline in their viability. This effect was both time- and concentration-dependent, with EA concentrations ranging from 10 to 30 µM and treatment durations ranging from 24 to 72 h. Additionally, the study showed that EA specifically and dose-dependently induced apoptosis in HeLa cells. The ability of EA to stop the cell cycle at the G1 phase was thought to be responsible for this apoptotic effect. EA accomplished this by changing the expression of proteins linked to the cell cycle, specifically cyclins, and by modifying the STAT3 signaling pathway [47]. Catechins, particularly those found in green tea, have shown anticancer properties via a variety of mechanisms, such as signaling pathway modulation and antioxidant activity [48]. Finally, at certain concentrations, GA has been shown to have pro-oxidant effects in cancer cells, which results in growth inhibition and apoptosis [49,50]. Our findings, along with those previously reported, indicate that the extract’s synergistic immunomodulatory and antioxidant effects are linked to its antitumoral effect. This suggests that the extract may have potential uses in cancer treatment through nutritional and phytotherapeutic approaches.

### 3.4. Anti-Diabetic Effect of Nitraria retusa Extract

**Figure 6:**
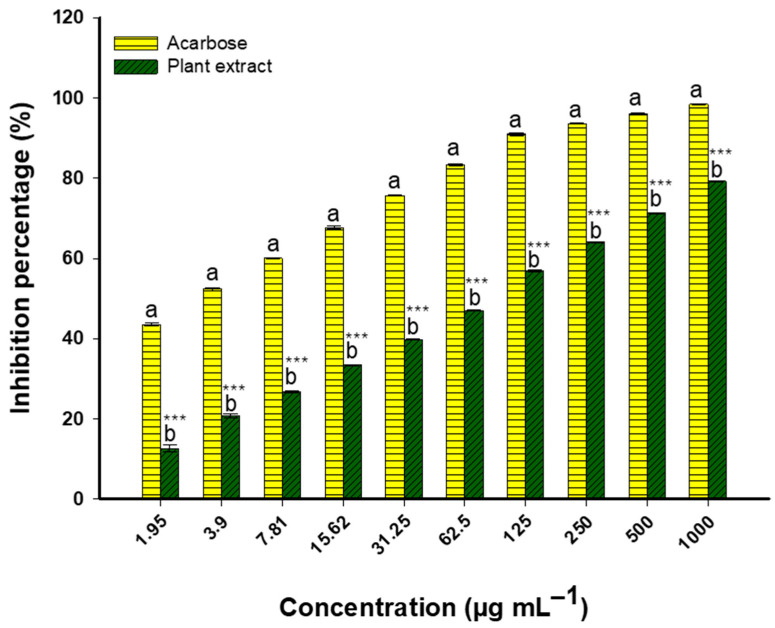
Dose-dependent α-amylase inhibition by *Nitraria retusa* extract vs. acarbose (1.95–1000 μg/mL). Different letters (a and b) on the bars at the same concentration indicate significant differences. *** *p* < 0.001 indicate significant differences from control group (*p* < 0.05, *p* < 0.01, and *p* < 0.001, respectively); (n = 3).

The potential anti-diabetic properties of *Nitraria retusa* extract were assessed using the α-amylase inhibition assay. The outcomes clearly showed that α-amylase activity was inhibited in a dose-dependent manner. Modest inhibition percentages of 12.6% and 20.6% were demonstrated by the extract at lower concentrations of 1.95 µg/mL and 3.9 µg/mL, respectively. The inhibitory effect increased with concentration: 31.25 µg/mL produced 39.7% inhibition, 62.5 µg/mL produced 46.9% inhibition, and 125 µg/mL produced 56.8% inhibition. Its IC_50_ was found to be 68.2 ± 4.2 µg/mL, greater than the IC_50_ of acarbose, the control (10.9 ± 1.52 µg/mL). At higher concentrations, the extract maintained its enhanced inhibitory activity, following the same pattern. Significant enzyme inhibition was observed in the extract at higher concentrations, specifically at 250 µg/mL (64.0%), 500 µg/mL (71.3%), and 1000 µg/mL (79.2%) (Figure 6).

**Figure 7:**
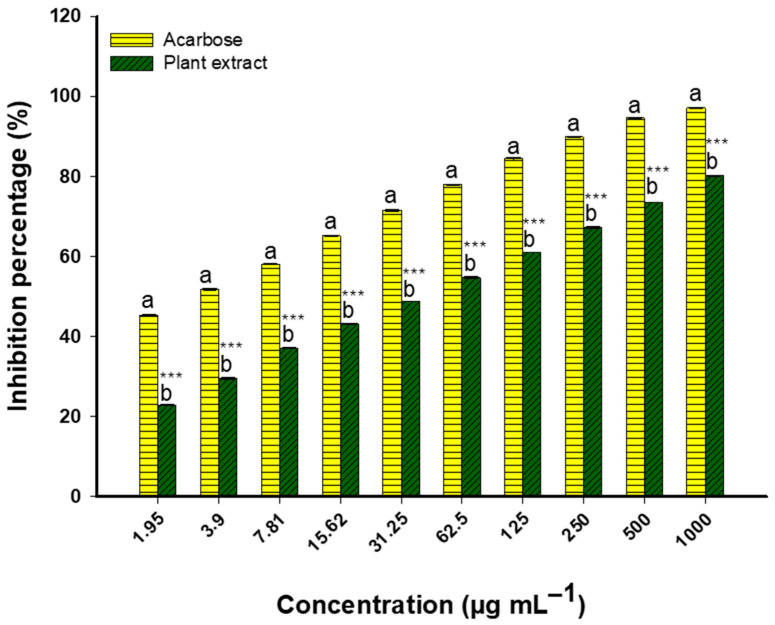
Profiles of glucosidase inhibition by plant extract and acarbose (1.95–1000 μg/mL. Different letters (a and b) on the bars at the same concentration indicate significant differences. *** *p* < 0.001 indicate significant differences from control group (*p* < 0.05, *p* < 0.01, and *p* < 0.001, respectively); (n = 3).

Regarding the α-glucosidase inhibition assay, the efficacy increased with increasing concentrations, indicating clear dose-dependent inhibition. The extract showed moderate inhibition at lower concentrations: 22.8% at 1.95 µg/mL and 29.5% at 3.9 µg/mL. The inhibitory effect increased with concentration, reaching 48.7% at 31.25 µg/mL, 61.0% at 125 µg/mL, and 67.2% at 250 µg/mL. Significant inhibition of enzyme activity was observed at the highest concentrations (73.5% at 500 µg/mL and 80.2% at 1000 µg/mL), suggesting potential anti-diabetic effects (Figure 7). Acarbose, the standard control, had an IC_50_ value of 15.9 ± 1.88 µg/mL, whereas the extract’s value was higher at 22.8 ± 3.36 µg/mL.

The presence of phenolics and flavonoids in *N. retusa* extract inhibits the activity of amylase and glucosidase enzymes, which decreases the breakdown of carbohydrates in the intestines, leading to lower glucose levels after eating [51]. Interestingly, oxidative stress, which is considered the main source of insulin resistance and dysfunction of β-cells, can be combated or alleviated by antioxidant substances such as catechin, ellagic acid, and gallic acid, which present in high quantities in *N. retusa* extract [22]. Finally, various published investigations have reported that bioactive constituents in plant extracts, such as flavonoids, phenolics, and alkaloids, can protect the cells from diabetes by improving insulin receptors, protecting β-cells from damage caused by oxidative stresses, and preventing the formation of Advanced Glycation End Products (AGEs) [52]. These AGEs are damaging substances formed as a result of non-enzymatic interaction between proteins/lipids and sugars, ultimately leading to diabetic complications and oxidative stress.

### 3.5. Nitraria retusa Antimicrobial Activity

**Figure 8:**
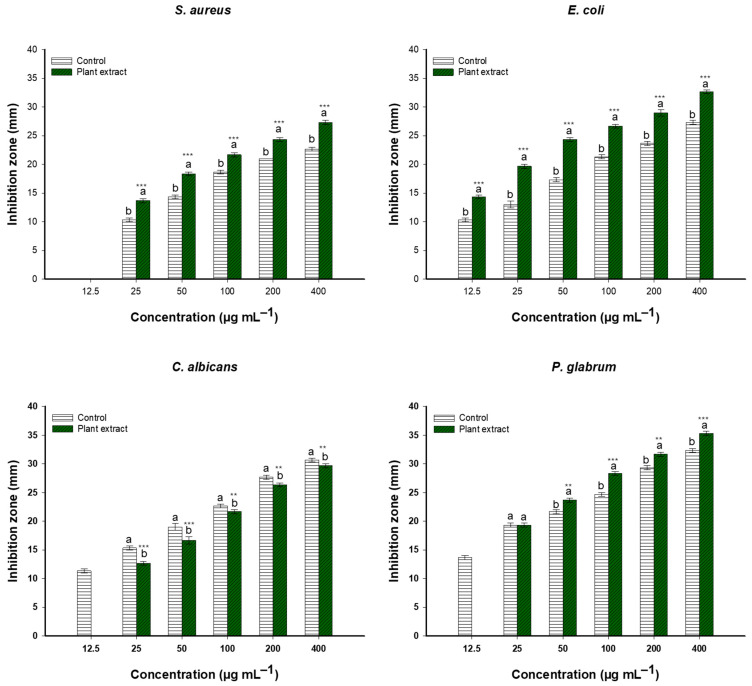
Antimicrobial potency represented as the inhibition zone (mm) of plant extract and control against *S. aureus*, *E. coli*, *C. albicans*, and *P. glabrum* across concentrations (12.5–400 μg/mL). Different letters (a and b) on the bars at the same concentration indicate significant differences. **, and *** *p* < 0.001 indicate significant differences from control group (*p* < 0.05, *p* < 0.01, and *p* < 0.001, respectively); (n = 3).

The extract’s antimicrobial activity was assessed against two bacterial strains (*S. aureus* and *E. coli*), a yeast (*Candida albicans*), and a fungal strain (*Penicillium glabrum*) using the agar well diffusion method. The negative control (DMSO) did not exhibit any antimicrobial activity, whereas the plant extract, as well as the positive control, exhibited a concentration-dependent inhibitory effect on the bacterial strains; *E. coli* exhibited the highest sensitivity, with inhibition zones ranging from 14.3 ± 0.6 mm at the lowest concentration (12.5 μg/mL) to 32.6 ± 0.6 mm at the highest concentration (400 μg/mL) vs. 10.3 ± 0.6 and 27.3 ± 0.6 mm for the gentamicin as positive control at the same above concentrations, respectively (Figure 8). On the other hand, *S. aureus* showed increasing zones from 13.6 ± 0.7 mm at 25 μg/mL to 27.3 ± 0.6 mm at 400 μg/mL but no inhibition at 12.5 μg/mL. Similarly, the positive control did not exhibit activity at the lowest concentration (12.5 μg/mL), and the activity increased from 10.3 12.5 ± 0.6 mm at 25 μg/mL to 22.7 ± 0.6 mm at 400 μg/mL. Interestingly, at all concentrations, the antibacterial activity of ethyl acetate plant extracts was superior to that of gentamicin. Moreover, *E. coli* consistently showed larger inhibition zones than *S. aureus*, indicating that the extract might be more effective against Gram-negative bacteria (Figure 8).

The effectiveness of ethyl acetate extracts against *Penicillium glabrum* and *Candida albicans* was observed at different levels. At the lowest concentration (12.5 μg/mL), the *N. retusa* extracts did not exhibit any activity, in contrast to fluconazole (positive control), which showed an inhibition zone of 11.3 ± 0.6 mm. By increasing the concentrations, the inhibition zones increased steadily, measuring 12.6 ± 0.6 mm at 25 μg/mL and 29.6 ± 0.6 mm at 400 μg/mL for the plant extract, whereas fluconazole showed inhibition activity, with values of 15.3 ± 0.6 mm at 25 μg/mL and 30.7 ± 0.6 mm at 400 μg/mL (Figure 8). *P. glabrum* showed greater sensitivity to the plant extract, exhibiting larger zones ranging from 19.3 ± 0.6 mm (vs. 19.2 mm for fluconazole) at 25 μg/mL to 35.3 ± 0.6 mm (vs. 32.3 ± 0.6 mm for fluconazole) at 400 μg/mL, but no inhibition at 12.5 μg/mL (vs. 13.7 ± 0.6 mm for positive control). Generally, fluconazole activity increased compared to plant extract activity against yeast, whereas multicellular fungi displayed larger inhibition zones upon being treated with *N. retusa* extract, especially at high concentrations, compared to the positive control (Figure 8).

HPLC analysis of the *N. retusa* extract’s phytochemical profile provides important information about its antimicrobial properties. The diverse range of bioactive compounds present in the extract implies that it has multiple mechanisms of action against different microorganisms, which explains the observed differential sensitivity among the tested microorganisms (*P. glabrum* > *C. albicans* > *S. aureus* > *E. coli*). The profile of the extract is dominated by the existing phenolic compounds, with high concentrations of gallic acid (1905.1 µg/g), ellagic acid (2671.1 µg/g), and catechin (1984.1 µg/g). These substances are well known for their wide range of antibacterial properties, which are mostly achieved by disrupting membranes. They cause increased permeability, the loss of cellular contents, and cell death when they interact with microbial cell membranes [53].

These phenolic substances could chelate the metal ions necessary for microbial growth and survival, as well as inhibit important microbial enzymes [54]. Significant concentrations of ferulic acid (782.4 µg/g) and vanillin (1083.5 µg/g), both recognized for their antimicrobial qualities, are also present in the extract. The antifungal properties of the extract against *Candida albicans* and *Candida glabrum* are primarily attributed to vanillin. These substances may function by obstructing quorum sensing in microbes or preventing the formation of biofilms, which would decrease their virulence and population growth [55,56].

Conversely, flavonoids, which include quercetin (14.39 µg/g), kaempferol (119.15 µg/g), and rutin (1010.0 µg/g), constitute another important class of compounds in the extract. Through a variety of methods, these flavonoids support overall antimicrobial activity. Their planar structure permits DNA intercalation, which may prevent transcription and microbial replication [57,58]. Furthermore, at high concentrations, certain flavonoids, such as quercetin, may have pro-oxidant properties that cause microbial cells to experience oxidative stress by producing reactive oxygen species [59].

The extract’s components, especially catechins (1984.1 µg/g), may have greater potency against Gram-negative bacteria, as evidenced by its greater effectiveness against *E. coli* when compared to *S. aureus*. This might be because catechins can obstruct the enzymes that Gram-negative bacteria use to synthesize their cell walls [60]. Additionally, certain substances derived from plants have the ability to block microbial efflux pumps, which raises the intracellular concentration and improves the effectiveness of antimicrobial substances [61]. Furthermore, the extract’s antimicrobial properties may be enhanced by specific phenolic compounds interfering with microbial fatty acid biosynthesis, which is essential for maintaining cell membrane integrity [40,62]. Synergistic antimicrobial effects are probably produced by the combination of these various bioactive compounds, which act on multiple targets at once to increase overall efficacy [63].

## 4. Conclusions

The extensive analysis of *Nitraria retusa* extract demonstrated that, owing to its rich polyphenolic profile, it possesses considerable antioxidant activity, outperforming ascorbic acid in DPPH tests. It showed minimal effect against normal cells and selective cytotoxicity against cancer cell lines, especially Caco2. It showed modest anti-diabetic properties, though less effective than standard treatments. Additionally, it showed broad-spectrum antimicrobial activity, especially against *E. coli* and *Penicillium glabrum*. All these results point to *Nitraria retusa* extract as a potentially useful source of bioactive compounds for antimicrobial interventions, antioxidant therapy, and cancer treatment. Although these findings are promising, more in vivo and molecular mechanistic research is required to completely clarify the extract’s safety profile, effectiveness, and potential benefits over conventional therapies. This will pave the way for the development of innovative plant-based therapeutic approaches.

## Figures and Tables

**Table 1:** Concentrations of the identified bioactive compounds separated from *Nitraria retusa* extract.

Identified Compound	RetTime [min]	Conc. (µg/g)	Identified Compound	RetTime [min]	Conc. (µg/g)
Gallic acid	3.586	1905.1	Vanillin	9.027	1083.5
Chlorogenic acid	4.250	0.00	Ferulic acid	9.332	782.4
Catechin	4.368	1984.1	Naringenin	10.397	27.3
Methyl gallate	5.345	65.2	Rosmarinic acid	11.751	110.8
Coffeic acid	5.921	284.9	Daidzein	15.831	4.06
Syringic acid	6.401	49.6	Querectin	17.210	14.3
Rutin	6.950	1010.0	Cinnamic acid	19.098	16.7
Ellagic acid	7.174	2671.1	Kaempferol	20.815	119.1
Coumaric acid	8.692	23.8	Hesperetin	21.396	52.7

## Data Availability

The data presented in this study are available on request from the corresponding author.

## Supplementary Materials

The following supporting information can be downloaded at: https://www.mdpi.com/article/10.3390/life14121629/s1, Figure S1: FT-IR spectrum of *Nitraria retusa* extract shows major functional groups, Table S1: Functional groups and bond types exhibited by the major FT-IR absorption peaks of *N. retusa* extract, Table S2: HPLC standards.
