# Supplementary material for: Phytochemical Analysis and Multifaceted Biomedical Activities of Nitraria retusa Extract as Natural Product-Based Therapies
Source: Life (Basel). 2024 Dec 9;14(12):1629. doi: 10.3390/life14121629 (PMC11676749; doi:10.3390/life14121629)
Supplement: Supplementary file 1 [file life-14-01629-s001.zip › life-3296222-supplementary.pdf]

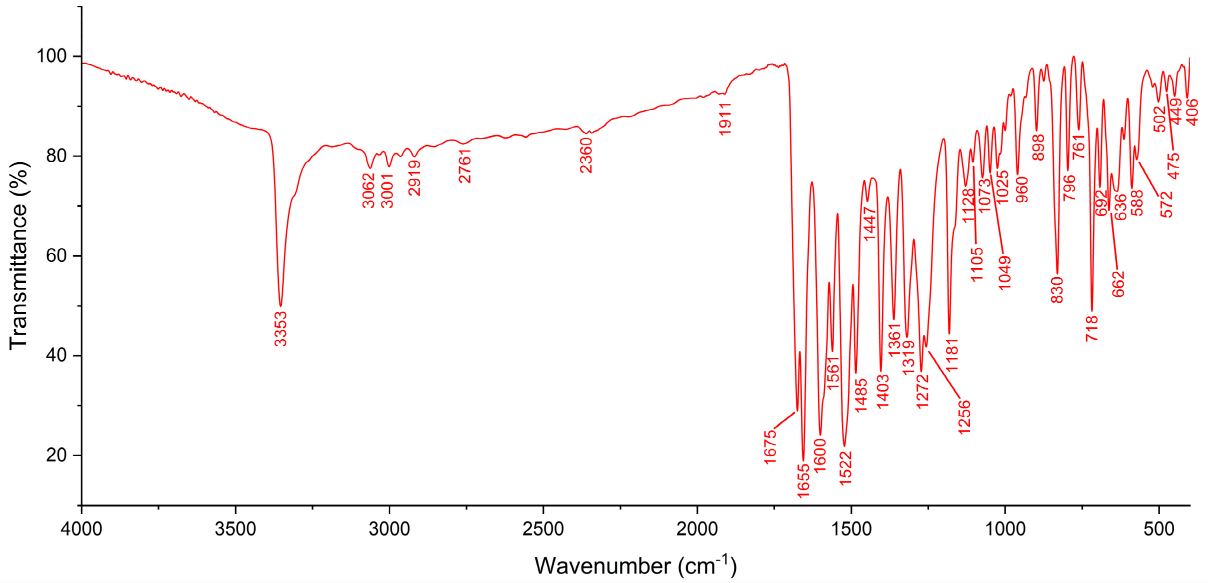

Figure S1. FT-IR spectrum of *Nitraria retusa* extract shows major functional groups.

Table S1. Functional groups and bond types exhibited by the major FT-IR absorption peaks of *N. retusa* extract.

Observed Peak (cm^{-1})	Functional Group	Bond Type	Characteristic Range (cm^{-1})	Relative Intensity
3353	Alcohol or Carboxylic Acid	O-H stretch	3200-3600	Strong
2919, 2851	Alkane	C-H stretch	2850-3000	Medium
1675	Carboxylic Acid	C=O stretch	1700-1725	Strong
1600	Aromatic Ring	C=C stretch	1450-1600	Medium
1485-1400	Alkane	C-H bend	1350-1480	Medium
1273	Carboxylic Acid	C-O stretch	1210-1320	Medium
1128	Alcohol	C-O stretch	1050-1150	Medium
1000-900	Alkene	=C-H bend	675-1000	Medium
720-630	Aromatic Ring	C-H bend	690-900	Weak

Table S2. HPLC Standards.

Standard	RetTime [min]	Area
Gallic acid	3.582	236.95
Chlorogenic acid	4.250	361.76
Catechin	4.480	316.29
Methyl gallate	5.494	278.17
Coffeic acid	5.928	210.48
Syringic acid	6.423	243.51
Rutin	6.931	292.69
Ellagic acid	7.256	732.00
Coumaric acid	8.703	556.21
Vanillin	9.103	330.27
Ferulic acid	9.752	334.60
Naringenin	10.436	314.22
Rosmarinic acid	11.936	466.58
Daidzein	16.040	299.82
Querectin	17.359	320.68
Cinnamic acid	19.268	533.02
Kaempferol	20.654	268.56
Hesperetin	21.232	402.53
